# Temporary Dietary Iron Restriction Affects the Process of Thrombus Resolution in a Rat Model of Deep Vein Thrombosis

**DOI:** 10.1371/journal.pone.0126611

**Published:** 2015-05-11

**Authors:** Makiko Oboshi, Yoshiro Naito, Hisashi Sawada, Shinichi Hirotani, Toshihiro Iwasaku, Yoshitaka Okuhara, Daisuke Morisawa, Akiyo Eguchi, Koichi Nishimura, Kenichi Fujii, Toshiaki Mano, Masaharu Ishihara, Tohru Masuyama

**Affiliations:** 1 Cardiovascular Division, Department of Internal Medicine, Hyogo College of Medicine, Nishinomiya, Japan; 2 Division of Coronary Heart Disease, Department of Internal Medicine, Hyogo College of Medicine, Nishinomiya, Japan; University of Florida, UNITED STATES

## Abstract

**Background:**

Deep vein thrombosis (DVT) is a major cause of pulmonary thromboembolism and sudden death. Thus, it is important to consider the pathophysiology of DVT. Recently, iron has been reported to be associated with thrombotic diseases. Hence, in this study, we investigate the effects of dietary iron restriction on the process of thrombus resolution in a rat model of DVT.

**Methods:**

We induced DVT in 8-week-old male Sprague-Dawley rats by performing ligations of their inferior venae cavae. The rats were then given either a normal diet (DVT group) or an iron-restricted diet (DVT+IR group). Thrombosed inferior venae cavae were harvested at 5 days after ligation.

**Results:**

The iron-restricted diet reduced venous thrombus size compared to the normal diet. Intrathrombotic collagen content was diminished in the DVT+IR group compared to the DVT group. In addition, intrathrombotic gene expression and the activity of matrix metalloproteinase-9 were increased in the DVT+IR group compared to the DVT group. Furthermore, the DVT+IR group had greater intrathrombotic neovascularization as well as higher gene expression levels of urokinase-type plasminogen activator and tissue-type plasminogen activator than the DVT group. The iron-restricted diet decreased intrathrombotic superoxide production compared to the normal diet.

**Conclusions:**

These results suggest that dietary iron restriction affects the process of thrombus resolution in DVT.

## Introduction

Deep vein thrombosis (DVT) involves the formation of blood clots in the deep veins of the body. DVT results from conditions that lead to stasis or turbulence of venous blood flow, endothelial damage, or hypercoagulability [1]. DVT mostly occurs in the lower legs or thighs and is the primary cause of pulmonary thromboembolism and sudden death [2]. Understanding of the pathophysiology of DVT is important for preventing its morbidity and mortality.

Iron is an important mineral needed for maintaining normal bodily functions. However, excess free iron leads to the production of hydroxyl radicals through the oxidation of its ferrous form to its ferric form through the Fenton reaction. Notably, iron is thought to be associated with the pathophysiology of several cardiovascular diseases [3,4]. In line with these findings, several epidemiologic studies have reported that body iron status is related to the risk of stroke and atherosclerosis [5,6]. Moreover, dietary iron intake has been reported to be positively correlated with mortality from stroke [7]. In addition, iron levels have been associated with the risk of acute myocardial infarction [8,9], and dietary iron intake has likewise been related to an increased risk of acute myocardial infarction [10,11]. These reports prompted us to explore the association of iron with thrombus in DVT.

When blood is exposed to iron, characteristic changes in thrombus are observed [12]. An experimental mouse model has shown that iron overload accelerates arterial thrombus formation [13]. However, to the best of our knowledge, there have been no investigations to determine whether dietary iron restriction impacts the process of thrombus resolution in DVT. Recently, we have reported that dietary iron restriction has preventive effects on salt-induced cardiovascular remodeling in Dahl salt-sensitive rats [14]. Therefore, we hypothesized that dietary iron reduction might affect the process of thrombus resolution in DVT. In this study, we investigate the effects of dietary iron restriction on the process of thrombus resolution in a rat model of stasis-induced thrombosis.

## Materials and Methods

### Ethics Statement

All of our experimental procedures were approved by the Animal Research Committee of Hyogo College of Medicine (protocol #13–036).

### Rat model of DVT

Seven-week-old male Sprague-Dawley rats were fed a normal diet for 1 week. Afterward, intravenous thrombosis was induced with proximal ligation of the inferior vena cava (IVC) below the renal veins under anesthesia with isoflurane, as previously described [15]. After IVC ligation, rats were randomly divided into two groups and were given a normal diet ([DVT] n = 6) or an iron-restricted (IR) diet ([DVT+IR] n = 6) for 9 days. The iron-restricted diet consisted of a normal diet with a mineral mixture free of FeC_6_H_5_O_7_∙5H_2_O as previously described [16]. Rats were maintained on a 12 hr light/dark cycle and had free access to food and water. Rats were sacrificed at 5 and 9 days after IVC ligation under anesthesia with isoflurane. The adequacy of anesthesia was confirmed as previously described [17]. The sacrificed rats’ blood was withdrawn by cardiac puncture, and the serum was stored at -80°C before analysis. Thrombosed IVC was then weighed and measured, quickly snap-frozen in liquid nitrogen, and stored at −80°C. Thrombus mass was defined as thrombus weight divided by thrombus vertical length as previously described [18,19], and thrombus volume was defined by multiplying thrombus cross-sectional area by thrombus vertical length as previously described [20]. Pieces of the thrombosed IVC tissue were placed in buffered 4% paraformaldehyde or embedded in Tissue-Tek OCT compound (Sakura Finetechnical Co.) and snap-frozen in liquid nitrogen.

### Assessments of Hematologic Parameters and Tissue Iron Content

Blood cell counts and serum iron concentrations were determined as previously described [16]. Tissue iron content of the thrombi was measured using a Metallo assay kit according to the manufacturer’s instructions (AKJ Global Technology, Chiba, Japan). Iron content was then corrected to thrombus weight for each sample.

### Histological Analysis

Thrombosed IVC tissues fixed with buffered 4% paraformaldehyde were embedded in paraffin and cut into 4-μm-thick sections. The paraffin-embedded sections were stained with hematoxylin-eosin (HE) and Masson’s trichrome. In addition, sections of paraffin-embedded tissue were immunohistochemically stained with anti-CD68 (AbD Serotec; dilution 1:1000) and von Willebrand Factor (vWF) antibodies (Dako; dilution 1:5000) and counterstained with hematoxylin. Immunostains were visualized with the use of an avidin-biotin-peroxidase conjugate and 3,3’-diaminobenzidine substrate. The number of CD68 positive cells or neovascular channels in 10 randomly selected high power fields (x1000) was counted in a blinded manner. In addition, thrombosed IVC tissues embedded in Tissue-Tek OCT compound were cut transversely into 10-μm thick cross-sections and incubated with dihydroethidium (DHE; 2 μmol/L, 37° C for 30 minutes; Sigma-Aldrich). NIH Image-J software was used to quantify the fibrosis area and DHE fluorescence intensity of the venous thrombus in the photomicrographs.

### Collagen Assays

The collagen content of the venous thrombi was measured using Sircol collagen assay kits according to the manufacturer’s instructions (Bicolor Ltd, Carrickfergus, United Kingdom). Collagen content was then corrected to thrombus weight for each sample.

### Gelatin Zymography

Matrix metalloproteinase-2 (MMP-2) and matrix metalloproteinase-9 (MMP-9) activity of the venous thrombi were determined by gelatin zymography. Gelatin zymography was performed with Gelatin Zymo-Electrophoresis Kits and MMP markers (Cosmo Bio Co.Ltd., Tokyo, Japan) according to the manufacturer’s instructions.

### Gene Expression Analysis

Total RNA was extracted from the venous thrombi using the TRIzol reagent (Invitrogen). The cDNA was prepared from DNase-treated RNA (Applied Biosystems). Real-time PCR was performed with ABI PRISM 7900 using TaqMan Universal PCR Master Mix and TaqMan Gene Expression Assays (Applied Biosystems) [17]. The following TaqMan Gene Expression Assays were used for each gene: assay ID Rn01538170_m1 for MMP-2, assay ID Rn00579162_m1 for MMP-9, assay ID Rn00565261_m1 for urokinase-type plasminogen activator (uPA), assay ID Rn01482578_m1 for tissue-type plasminogen activator (tPA), assay ID Rn01481341_m1 for plasminogen activator inhibitor-1 (PAI-1), assay ID Rn1462586_m1 for fibrinogen α chain, and assay ID Rn99999916_s1 for glyceraldehyde-3-phosphate dehydrogenase (GAPDH). GAPDH was used as an internal control.

### Western blot Analysis

Thrombus tissues were prepared in ice-cold lysis buffer containing 1 mM PMSF and separated using SDS-PAGE. An ECL Western blotting Detection kit (Thermo Scientific) was used for detection, and the images were analyzed using Image Quant LAS 4000 mini (GE Health Care). Antibodies against CD31 (AbD Serotec; dilution 1:1000), transferrin receptor 1 (TfR1; Zymed Laboratories, dilution 1: 1000), and rabbit anti-GAPDH (Cell Signaling Technology, dilution 1: 1000) were used in this study.

### Statistical Analysis

Values are reported as the means ± SD. Statistical analysis was performed using Student’s *t* test. A p value <0.05 denoted the presence of a statistically significant difference.

## Results

### Dietary Iron Restriction Affected Stasis-Induced Venous Thrombus in Rats

**Fig 1:**
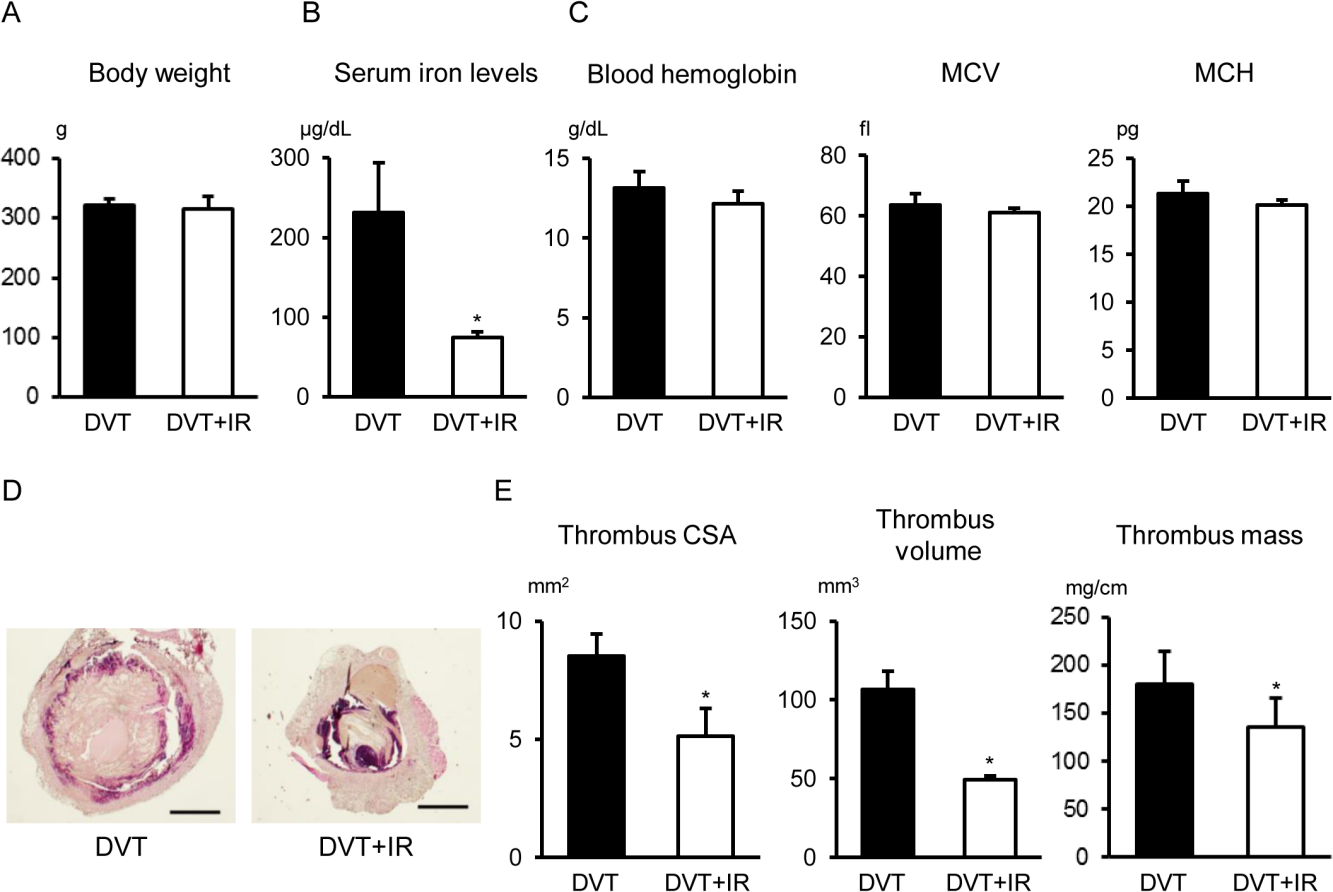
Effects of Temporary Dietary Iron Restriction on Stasis-Induced Venous Thrombi in Rats. (A) Body weight, (B) Serum iron levels, (C) blood hemoglobin content, mean corpuscular volume (MCV), and mean corpuscular hemoglobin (MCH) in the DVT and DVT+IR groups (n = 6 in each group). (D) Representative images of hematoxylin-eosin staining of the venous thrombus. Scale bars: 1 mm. (E) Thrombus cross-sectional area (CSA), thrombus volume, and thrombus mass in the DVT and DVT+IR groups (n = 5 in each group). DVT, rats with IVC ligation fed a normal diet; DVT+IR, rats with IVC ligation fed an iron-restricted diet. *p < 0.05 versus the DVT group.

First, we evaluated the effects of dietary iron restriction on physiological parameters. Body weight was not different between the groups at 5 days after IVC ligation (Fig 1A). Dietary iron restriction reduced serum iron levels at 5 days after IVC ligation but caused no change in blood hemoglobin content, mean corpuscular volume, or mean corpuscular hemoglobin (Fig 1B and 1C). Next, we assessed whether dietary iron reduction affects stasis-induced venous thrombosis in rats. HE staining showed that the venous thrombi developed in rats at 5 days after IVC ligation (Fig 1D). Compared to the DVT group, venous thrombus size was attenuated in the DVT+IR group (Fig 1D). This was consistent even if the thrombi were measured by thrombus cross-sectional area, thrombus volume, and thrombus mass (Fig 1E). The attenuation in venous thrombus size was also observed at 9 days after IVC ligation (thrombus cross-sectional area; 3.0 ± 0.8 vs 1.4 ± 0.3 mm^2^, thrombus volume; 20.0 ± 5.4 vs 10.2 ± 2.6 mm^3^, thrombus mass; 70.0 ± 6.9 vs 53.6 ± 5.7 mg/cm; DVT vs DVT+IR, p<0.05). These results suggest that dietary iron restriction affects stasis-induced venous thrombosis in rats.

### Dietary Iron Restriction Accelerated the Resolution of Stasis-Induced Venous Thrombus in Rats

**Fig 2:**
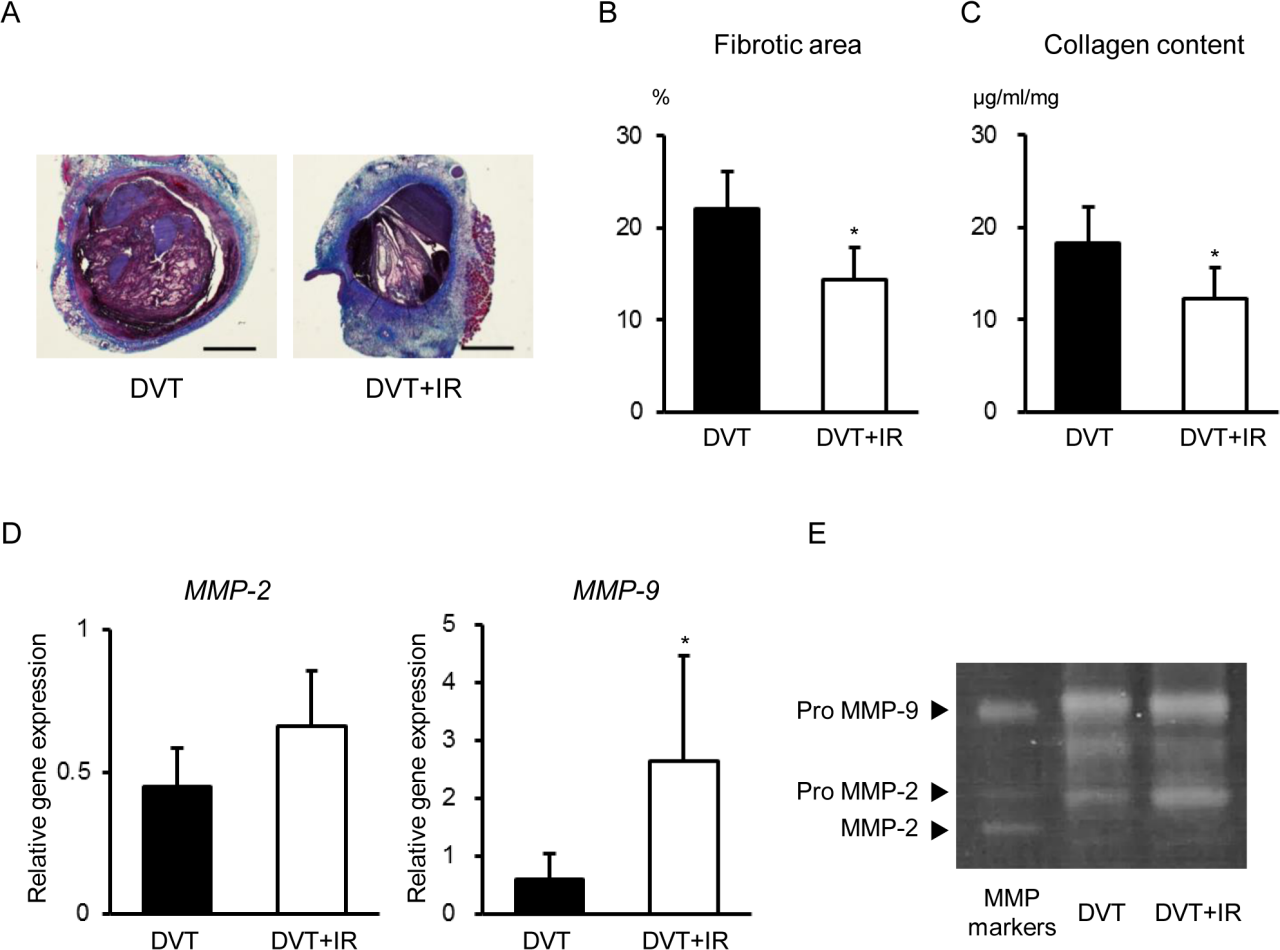
Effects of Temporary Dietary Iron Restriction on Collagen Contents of Stasis-Induced Venous Thrombi in Rats. (A) Representative images of Masson’s trichrome staining of the venous thrombi. Scale bars: 1 mm. (B) Quantification of fibrosis area and (C) collagen content assessed by collagen assays in the venous thrombi of the DVT and DVT+IR groups (n = 6 in each group). Intrathrombotic (D) gene expression and (E) enzymatic activity of MMP-2 and MMP-9 in the DVT and DVT+IR groups (n = 4 in each group). *p < 0.05 versus the DVT group.

Because intrathrombotic collagen content can affect venous thrombus size, we next examined intrathrombotic collagen contents in these animals. Masson’s trichrome staining showed that an increased intrathrombotic collagen area was observed at 5 days after IVC ligation. Compared with the DVT group, the intrathrombotic collagen area was diminished in the DVT+IR group (Fig 2A and 2B). Likewise, intrathrombotic collagen content was decreased in the DVT+IR group compared to the DVT group (Fig 2C).

MMP-2 and MMP-9 play critical roles in collagenolysis during venous thrombus resolution [18]. Thus, we assessed intrathrombotic gene expression and enzymatic activity of MMP-2 and MMP-9 in our model system. Intrathrombotic gene expression of MMP-2 tended to be increased in the DVT+IR group at 5 days after IVC ligation compared to the DVT group and (Fig 2D). In addition, intrathrombotic gene expression of MMP-9 was increased in the DVT+IR group at 5 days after IVC ligation compared to the DVT group (Fig 2D). Moreover, intrathrombotic enzymatic activities of MMP-2 and MMP-9 were increased in the DVT+IR group compared to the DVT group (Fig 2E). These results suggest that dietary iron restriction affects venous thrombus resolution by increasing intrathrombotic enzymatic activities of MMP-2 and MMP-9.

### Dietary Iron Restriction Affected Intrathrombotic Neovascularization and Plasmin System but not Leukocyte Recruitment of Stasis-Induced Venous Thrombus in Rats

**Fig 3:**
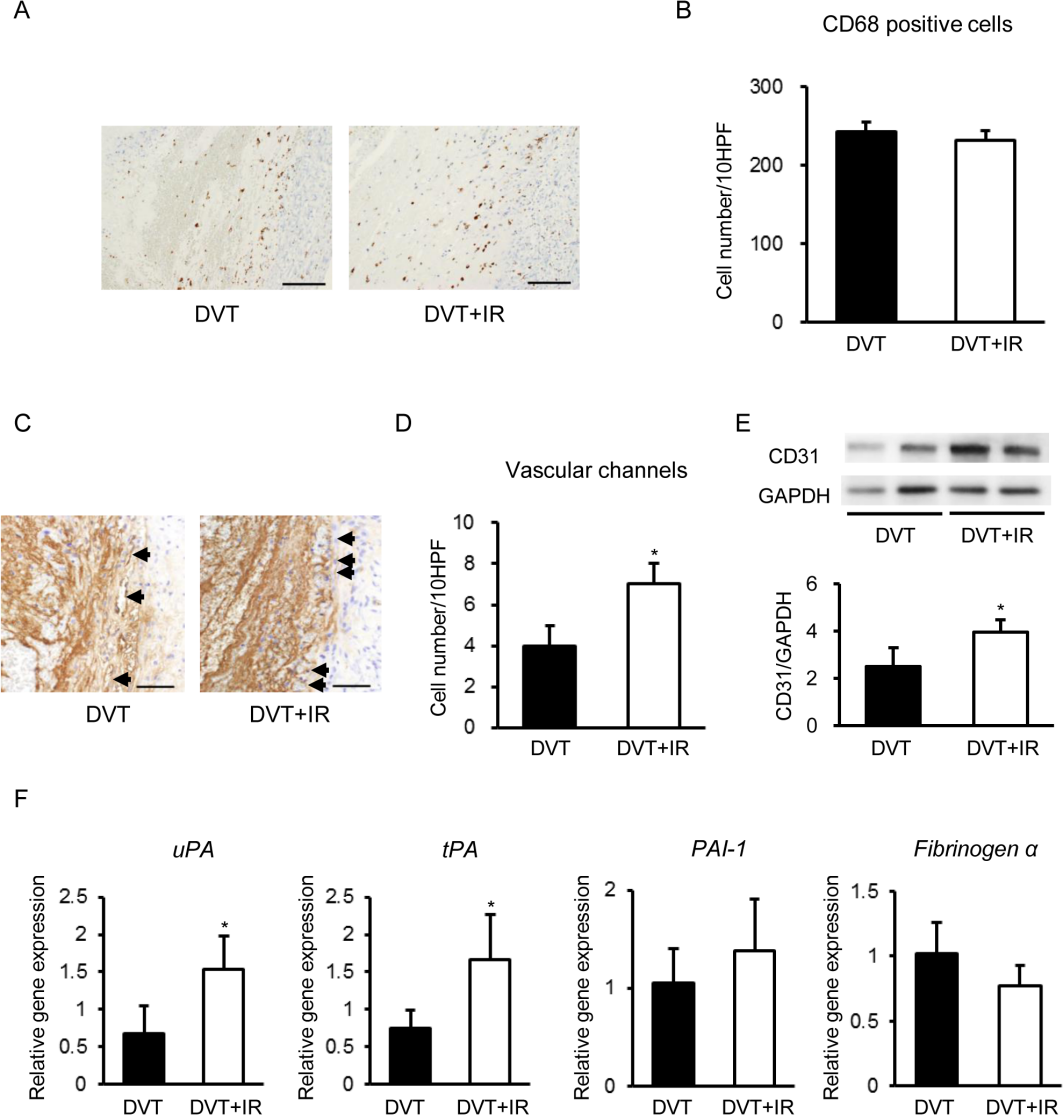
Effects of Temporary Dietary Iron Restriction on Leukocyte Recruitment, Neovascularization, and Plasmin System of Stasis-Induced Venous Thrombi in Rats. (A) Representative images of CD68 staining of the venous thrombi. Scale bars: 100 μm. (B) Quantification of CD68 positive cells in the venous thrombi of the DVT and DVT+IR groups (n = 6 in each group). (C) Representative images of vWF staining of the venous thrombi. Scale bars: 50 μm. Arrows show vascular channels. (D) Quantification of vascular channels in the venous thrombi of the DVT and DVT+IR groups (n = 6 in each group). The number of CD68 positive cells or vascular channels in 10 randomly selected high power fields (HPF) was counted in a blinded manner. (E) CD31 protein expression (top; representative gel blots depicting expression of CD31 and GAPDH, bottom; relative levels of expression) in the venous thrombi of the DVT and DVT+IR groups (n = 5 in each group). (F) Intrathrombotic gene expression of *uPA*, *tPA*, *PAI-1*, and *fibrinogen α chain* in the DVT and DVT+IR groups (n = 4 in each group). *p < 0.05 versus the DVT group.

As intrathrombotic leukocytes are reported to contribute to venous thrombus resolution [18,21], we next evaluated intrathrombotic leukocyte recruitment in these animals. The amount of thrombus-infiltrating CD68-positive macrophages were not different between the groups (Fig 3A and 3B).

Because intrathrombotic neovascularization also plays a role in venous thrombus resolution [22], we next examined intrathrombotic neovascularization in these groups. The number of intrathrombotic vWF-positive vascular channels was increased in the DVT+IR group at 5 days after IVC ligation compared to the DVT group (Fig 3C and 3D). Intrathrombotic CD31 expression was also increased in the DVT+IR group compared to the DVT group (Fig 3E).

Additionally, the plasmin system is associated with cell migration and subsequent thrombus resolution [23]. We assessed intrathrombotic gene expression of uPA, tPA, and PAI-1 in these groups. Intrathrombotic gene expression levels of uPA and tPA were increased in the DVT+IR group at 5 days after IVC ligation compared to the DVT group (Fig 3F). On the other hand, intrathrombotic gene expression of PAI-1 did not differ between the groups (Fig 3F). Although iron has been reported to bind to fibrinogen [12], intrathrombotic gene expression of fibrinogen α chain was not different between the groups (Fig 3F).

### Dietary Iron Restriction Affected Intrathrombotic Superoxide Production of Stasis-Induced Venous Thrombus in Rats

**Fig 4:**
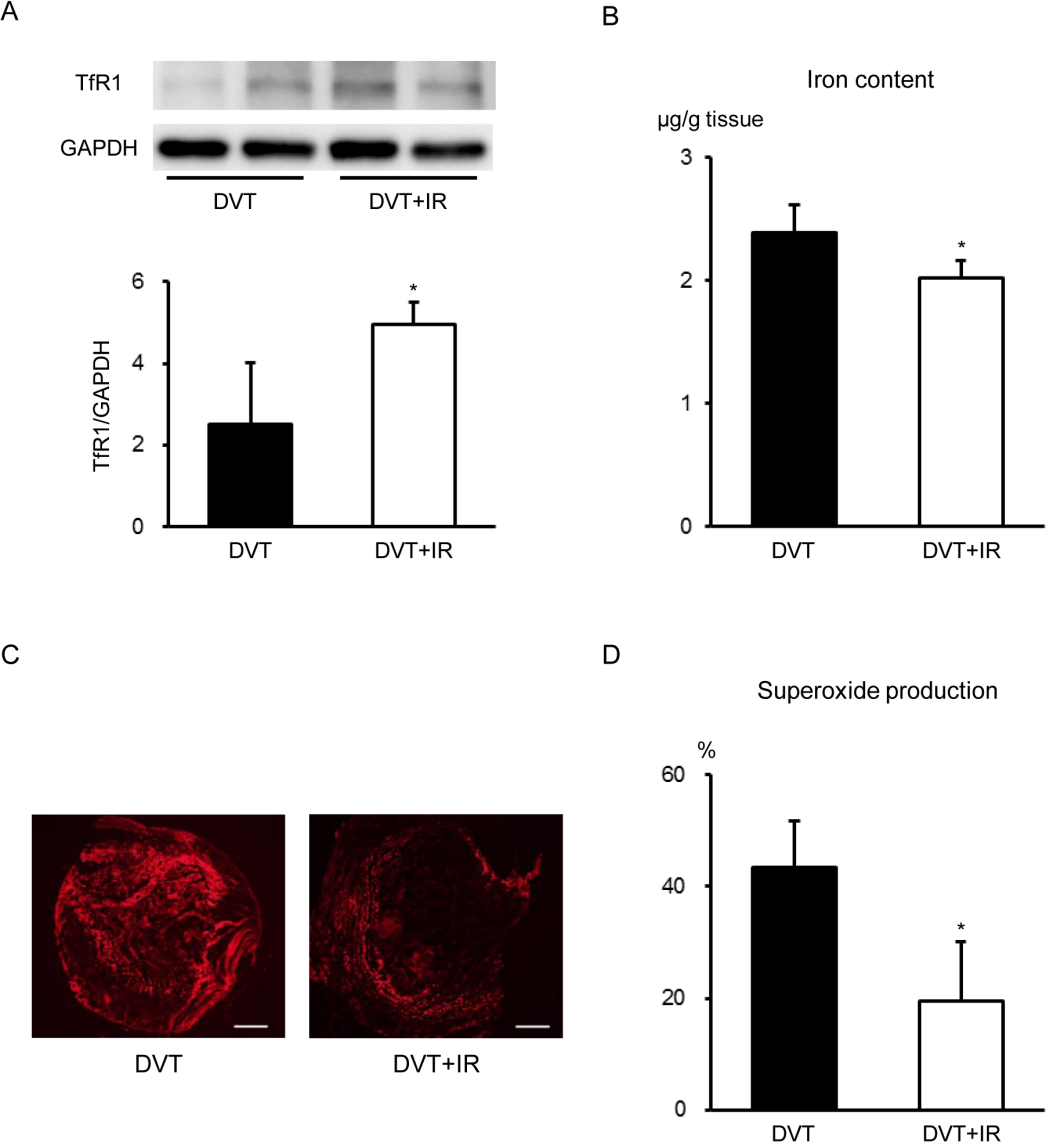
Effects of Temporary Dietary Iron Restriction on Oxidative Stress of Stasis-Induced Venous Thrombi in Rats. (A) TfR1 protein expression (top; representative gel blots depicting expression of TfR1 and GAPDH, bottom; relative levels of expression) and (B) iron content in the venous thrombi of the DVT and DVT+IR groups (n = 5 in each group). (C) Representative images of DHE staining of the venous thrombi. Scale bars: 500 μm. (D) Quantification of superoxide production in the venous thrombi of the DVT and DVT+IR groups (n = 5 in each group). *p < 0.05 versus the DVT group.

Next, to investigate whether cellular iron transport and iron are associated with the physiopathology of venous thrombosis, we examined intrathrombotic expression of TfR1, an intracellular iron transport protein and intrathrombotic iron contents in these groups. TfR1 is a membrane protein that mediates uptake of transferrin-bound iron into cells [24]. We found that TfR1 was expressed in the venous thrombi (Fig 4A). Compared to the DVT group, intrathrombotic TfR1 expression was increased in the DVT+IR group (Fig 4A). However, intrathrombotic iron content was decreased in the DVT+IR group compared to the DVT group (Fig 4B), indicating that intrathrombotic TfR1 expression is increased in response to iron deficiency in the DVT+IR group.

Oxidative stress plays an important role in the physiopathology of venous thrombosis [25,26]. Thus, we examined intrathrombotic superoxide production in these animals by DHE staining. Intrathrombotic superoxide production was significantly attenuated in the DVT+IR group compared to the DVT group (Fig 4C and 4D).

## Discussion

To the best of our knowledge, this is the first report to demonstrate the impact of dietary iron restriction on the process of thrombus resolution in a rat model of DVT.

Iron is an important mineral needed for maintaining normal bodily functions. However, excess free iron leads to the production of hydroxyl radicals through the oxidation of its ferrous form to its ferric form through the Fenton reaction. Notably, iron has been associated with the pathophysiology of several cardiovascular diseases [3,4]. Likewise, several epidemiologic studies have reported that body iron status is related to the risk of stroke and acute myocardial infarction [5,6,8,9]. Moreover, dietary iron intake has been reported to be associated with increased mortality from stroke and risk of acute myocardial infarction [7,10,11]. In this study, we elucidated the impact of dietary iron restriction on the process of thrombus resolution in a rat model of DVT. We found that temporary dietary iron restriction attenuated stasis-induced venous thrombus size in rats. Indeed, iron has been reported to enhance coagulation and attenuate fibrinolysis as documented by spectrophotometric and scanning electron micrographic techniques [27,28]. Similarly, dietary iron restriction seems to affect coagulation and fibrinolysis in our rat model of DVT.

Iron is associated with oxidative stress and characteristic changes in thrombus [13,27,28]. In addition, clinical studies have reported that oxidative stress plays a role in the physiopathology of venous thrombosis [25,26]. Our study is the first to observe an increased production of superoxide within the venous thrombi in rats and that this intrathrombotic superoxide production is attenuated by iron restriction. These data suggest that dietary iron restriction may accelerate venous thrombus resolution through inhibition of oxidative stress. Oxidative stress is associated with collagen production. On the other hand, MMP-2 and MMP-9 play roles in collagenolysis during venous thrombus resolution [18]. We observed reduced intrathrombotic superoxide production and increased enzymatic activities of MMP-2 and MMP-9 in the DVT+IR group compared to the DVT group. Therefore, both decreased oxidative stress and increased enzymatic activities of MMP-2 and MMP-9 may contribute to decreased collagen content in the DVT+IR groups.

Intrathrombotic macrophage/monocyte recruitment has been reported to contribute to venous thrombus resolution [18,21]. In this study, dietary iron restriction did not change the number of intrathrombotic CD68-positive macrophages. Therefore, the effects of dietary iron restriction on venous thrombus resolution are not likely to depend on leukocyte recruitment and/or chemokine expression. Intrathrombotic neovascularization also plays a role in venous thrombus resolution [22]. In addition, the plasmin system is associated with subsequent thrombus resolution [23]. In this study, the DVT+IR group had a greater number of intrathrombotic vascular channels and higher intrathrombotic gene expression of uPA and tPA than the DVT group. Thus, it is likely that dietary iron restriction accelerates venous thrombus resolution by increasing intrathrombotic neovascularization and gene expression of uPA and tPA. On the other hand, MMP-9 is reported to show proangiogenic activity by mediating basement membrane remodeling for endothelialization of tissue matrix [29]. Therefore, increased MMP-9 could promote intrathrombotic neovascularization. However, the particular mechanisms by which iron restriction affects intrathrombotic neovascularization, uPA and tPA gene expression, and MMP-9 activity remain unknown. Future experimental work will define the mechanism how dietary iron restriction accelerates thrombus resolution in DVT.

Because DVT sometimes occurs without any symptoms, it is difficult to prevent and diagnose at an early stage. DVT remains the primary cause of pulmonary thromboembolism and sudden death [2]. Therefore, it is important to diagnose DVT at an early stage to prevent pulmonary thromboembolism. Although it has been reported that severe iron deficiency anemia is associated with thrombosis [30–32], our rat model of DVT shows that temporary iron restriction may have beneficial effects on the process of thrombus resolution. These differences observed in our study compared to previous studies [30–32] may be due to the differences in the extent of iron deficiency. Temporary dietary iron restriction reduced serum iron levels but did not induce anemia. Severe iron deficiency and the resultant anemia are certainly not good for the body. Although future studies are necessary to determine the suitable levels of iron in the body for accelerating the process of thrombus resolution of DVT, these results suggest that temporary dietary iron restriction may be a novel therapeutic strategy for thrombus resolution in DVT.

In conclusion, ours is the first study showing that dietary iron restriction affects the process of thrombus resolution in a rat model of DVT. The beneficial effects of iron restriction on thrombosis seem to be associated with inhibition of oxidative stress. Temporary dietary iron restriction may lead a novel therapeutic strategy for thrombus resolution in DVT.
